# Explaining Gender Differences in Depressive Symptoms Among Caregivers of Older Patients With Critical Illness

**DOI:** 10.1093/geroni/igab046.1471

**Published:** 2021-12-17

**Authors:** Wenhao Fu, Jiajia Li

**Affiliations:** Shandong University, Jinan, Shandong, China (People's Republic)

## Abstract

The aging of baby boomers makes caring for the elderly an increasingly important topic. As rising cost of health care, the care for seriously ill patients has gradually shifted from hospitals to families, particularly in the countryside. Along with growing demand for informal care, informal caregivers are at increased risk of depression. The aim of this study was to explore the potential protective factors or risk factors associated with depressive symptoms of caregivers for patients with critical illness (45 to 93 years of age) across gender groups, explain their different pathways of influence, and elucidate targeted measures to improve their outcomes (N=518). Results from the statistical model showed that the paths of effect from care needs to caregiver depressive symptoms differed between male and female informal caregivers. Care needs were not significantly associated with depression symptoms among informal caregivers, for either men or women. Care hours of more than 12 hours per day and financial difficulties at home are risk factors for depressive symptoms in caregivers, with significance of OR=3.42; 95%CI,1.97 to 5.94; P=0.000 and OR=2.98; 95%CI, 1.46 to 6.05; p=0.003, respectively. For male caregivers, years of caregiver education and the feel relied upon by relative's were both protective factors, whereas Job-Caregiving conflict, was a risk factor (P<0.05). For female caregivers, caregiver burden and higher caregiver age were its risk factors (P<0.05). These important findings demonstrate that to be effective in reducing depressive symptoms among informal caregivers, both cointerventions and triage interventions by gender are warranted.

